# A Case of Acquired Hemophilia A Following Robot-Assisted Minimally Invasive Esophagectomy

**DOI:** 10.70352/scrj.cr.25-0797

**Published:** 2026-03-04

**Authors:** Shota Eguchi, Yoshio Nagahisa, Kenji Yamaguchi, Yukio Inamura, Michio Okabe, Toshihiko Masui

**Affiliations:** Department of General Surgery, Kurashiki Central Hospital, Kurashiki, Okayama, Japan

**Keywords:** esophageal cancer, robot-assisted surgery, acquired hemophilia A (AHA), immune checkpoint inhibitors (ICIs)

## Abstract

**INTRODUCTION:**

Acquired hemophilia A (AHA) is a rare but potentially fatal bleeding disorder that arises suddenly in individuals without a prior history of bleeding tendency. It is often associated with malignant disease and can present with severe hemorrhagic complications. Reports of AHA occurring in the postoperative course of esophageal cancer surgery are extremely limited, and the safety of immune checkpoint inhibitors (ICIs) in patients with a history of AHA remains uncertain. This case highlights both the diagnostic and therapeutic challenges of AHA and provides novel insight into the safe administration of ICIs in a patient with recurrent esophageal cancer following remission of AHA.

**CASE PRESENTATION:**

We describe a 77-year-old man with clinical stage II esophageal cancer who received neoadjuvant chemotherapy with docetaxel, cisplatin, and fluorouracil, followed by robot-assisted thoracoscopic and laparoscopic subtotal esophagectomy. Postoperatively, he developed an anastomotic stricture requiring re-anastomosis via median sternotomy. After the second surgery, he experienced uncontrollable wound bleeding, and laboratory testing revealed a markedly prolonged activated partial thromboplastin time. Further evaluation confirmed the diagnosis of AHA, for which medical therapy was initiated, resulting in remission. Subsequently, the patient developed recurrent esophageal cancer and is currently receiving fluorouracil plus cisplatin in combination with pembrolizumab. Notably, no relapse of AHA has been observed during immunotherapy.

**CONCLUSIONS:**

This case illustrates that AHA can complicate the postoperative course of esophageal cancer surgery, leading to significant bleeding difficulties. It also suggests that ICIs may be administered relatively safely in patients with recurrent esophageal cancer who have previously developed AHA. Clinicians should remain vigilant for this rare but serious condition when encountering unexplained bleeding in cancer patients, and the findings may provide reassurance regarding the use of immunotherapy in similar clinical contexts.

## Abbreviations

AHA
: acquired hemophilia A
APTT
: activated partial thromboplastin time
ICI
: immune checkpoint inhibitors
PT
: prothrombin time

## INTRODUCTION

AHA is caused by autoantibodies against factor VIII and is relatively rare, with an estimated incidence of 1–4 cases per million population.^1)^ The mortality rate has been reported to range from 8% to 22%, with most deaths occurring in the first few weeks after its presentation.^1,2)^ The majority of cases are idiopathic (43.6%–51.9%), but some cases occur with malignancy (6.4%–18.4%), and autoimmune disorders (9.4%–17.0%).^3)^ We present a case of AHA that developed following robot-assisted subtotal esophagectomy. The patient subsequently experienced postoperative recurrence of esophageal cancer and was treated with fluorouracil and cisplatin in combination with pembrolizumab, without relapse of AHA. To our knowledge, this is the first reported case in which ICI was administered for postoperative recurrence of esophageal cancer in a patient with AHA.

## CASE PRESENTATION

A 77-year-old man underwent a routine examination that revealed findings consistent with esophageal squamous cell carcinoma. His past medical history included carotid artery thrombosis treated with aspirin, hypertension, and hyperuricemia. He had previously undergone an appendectomy several decades earlier without complications. Before chemotherapy, the TNM classification was T2N0M0, corresponding to cStage II. The patient received two courses of neoadjuvant chemotherapy with DCF (docetaxel, cisplatin, and 5-fluorouracil), followed by robot-assisted subtotal esophagectomy. Reconstruction was performed using a retrosternal gastric tube, and a cervical esophagogastric anastomosis was performed using a powered circular stapler. The postoperative pathological findings showed ypT1aN0M0, corresponding to ypStage0 , and the histological treatment response was Grade 2. The operative time was 414 minutes, and intraoperative blood loss was approximately 30 mL. His postoperative course was uneventful, and he was discharged on POD 35 without pleural effusion.

**Fig. 1:**
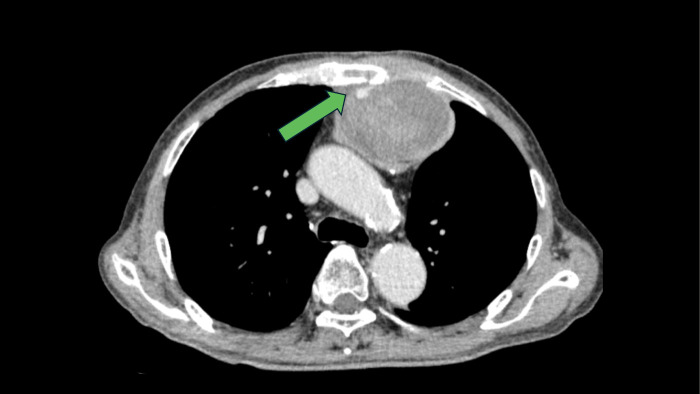
Hematoma located in the area of sternal separation The green arrow indicates the site of sternal dehiscence, posterior to which a gradually enlarging hematoma was observed. Active bleeding could not be ruled out.

Postoperatively, the patient developed recurrent anastomotic stricture. Despite three sessions of endoscopic balloon dilatation and radial incision cutting under general anesthesia, granulation tissue formed soon after each procedure, resulting in relapse. Consequently, re-anastomosis via sternotomy was performed on POD 171. The cervical approach was performed through a Y-shaped incision, and the stenotic segment of the retrosternal gastric tube was identified by dissecting along its course. The stricture was treated by a longitudinal incision followed by transverse closure, and drains were placed anterior and posterior to the repaired gastric tube. The operative time was 237 minutes, with intraoperative blood loss of approximately 540 mL. A drain was placed, but bloody discharge persisted. Laboratory data revealed anemia (hematocrit 26.7%), prolonged activated partial thromboplastin time (APTT) (56.9 s), and normal prothrombin time (PT) (13.6 s). Factor VIII activity was markedly reduced (9.9%), while the activities of other clotting factors were normal. The factor VIII inhibitor was measured at 1.7 Bethesda units/mL, and an APTT cross-mixing test demonstrated a pattern consistent with an inhibitor. A hematoma was also noted anterior to the sternum (**Fig. 1**). Based on the clinical course and laboratory findings, AHA was diagnosed.

**Fig. 2:**
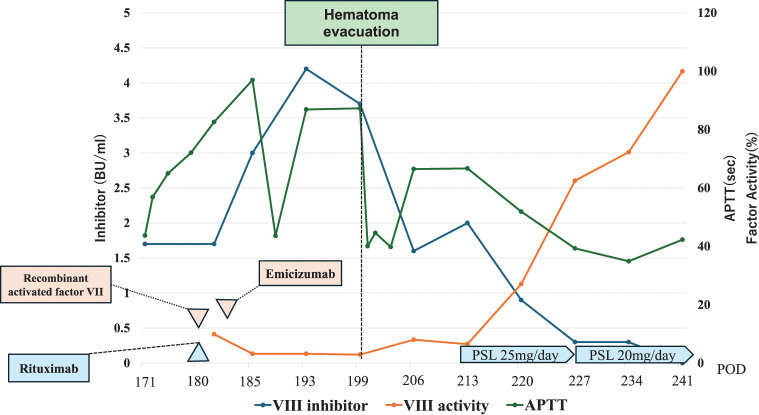
Time course of inhibitor and APTT treatment was initiated on day 9 after onset, during which frequent transfusions were required. Following the initiation of therapy, the inhibitor showed a decreasing trend.

Given concerns about wound infection and anastomotic leakage, treatment with rituximab and recombinant activated factor VII (NovoSeven) was initiated. However, the anterior hematoma did not improve, and hematoma removal surgery was performed on POD 199. As the preoperative APTT was markedly prolonged at 87.3 seconds, perioperative hemostatic management was planned in consultation with the hematology team. In addition to securing blood products before surgery, NovoSeven was scheduled for postoperative administration under hematology supervision. The reoperation was performed through the previous cervical incision. The hematoma was evacuated and the cavity was irrigated. The entire wound surface was coagulated, and adequate hemostasis was confirmed. A topical hemostatic agent (microporous polysaccharide hemospheres; Arista) was then applied. Two drains were placed in the hematoma cavity, and the incision was closed with mattress sutures. Postoperatively, NovoSeven was administered as planned for 3 days, after which hemostatic therapy was transitioned to emicizumab. The procedure lasted 73 minutes, with minimal blood loss, and no postoperative bleeding was observed. Hemostasis was considered well controlled, and emicizumab therapy was subsequently introduced. The inhibitor gradually declined over time. Steroid therapy was then initiated, resulting in normalization of APTT and restoration of factor VIII activity. Steroids were successfully tapered, and remission was achieved on POD 279 (corresponding to day 109 since onset) (**Fig. 2**).

At 1 year and 6 months after esophagectomy, pulmonary metastasis and metastasis to the right supraclavicular lymph node were detected, and treatment with FP plus pembrolizumab was initiated. While continuing follow-up with the hematology department, the patient received cisplatin (80 mg/m^2^ IV on day 1), continuous 5-fluorouracil (800 mg/m^2^/day on days 1–5), and pembrolizumab (200 mg IV on day 1). Before initiating therapy, factor VIII levels were preserved, no inhibitors were detected, and the APTT (33.4 s) was within the normal range. One week after treatment initiation, the APTT remained normal (29.4 s), and laboratory tests performed near the end of the first cycle also showed no abnormalities in APTT, factor VIII levels, or inhibitor assays. Pembrolizumab has since been administered for nine courses over 4 months, and similar monitoring during subsequent cycles revealed no prolongation of APTT or recurrence of AHA. Throughout this period, the tumor remained controlled without evidence of progression, and no irAEs or other adverse events were observed.

## DISCUSSION

AHA is a rare bleeding disorder caused by autoantibodies that neutralize coagulation factor VIII. It is associated with underlying conditions such as pregnancy, autoimmune diseases, malignancies, and drug reactions.^4)^ The clinical impact of AHA is higher because the severity of bleeding is affected by diagnostic delays and inadequate treatment.^5)^ Awareness of all specialists, especially surgeons, for this unexpected life-threatening bleeding episode is crucial.

A UK surveillance study reported that 37% (55/150) of AHA patients had an underlying condition, of which malignancy was documented in 40% (22/55).^6)^ In another review, Sallah et al.^7)^ described 41 cancer patients with AHA, including 25 (61%) with solid tumors and 16 (39%) with hematologic malignancies. Collectively, these findings suggest that malignancy is an important predisposing factor for the development of AHA. Nevertheless, despite this association, our literature search (performed on December 3, 2025, using the key words “acquired hemophilia A,” “factor VIII inhibitor,” “cancer,” “esophageal cancer,” and “surgery”) identified only one reported case of acquired hemophilia following esophageal cancer surgery.^8)^ By contrast, numerous reports describe surgery-associated AHA triggered by surgical trauma and perioperative immune dysregulation, even in patients without malignancy.^9)^ In our case as well, the temporal relationship strongly suggests that surgical stress, including reoperation, may have contributed to the development of AHA.

In general, AHA is characterized by normal bleeding time, platelet count, and PT, with prolongation observed only in APTT. When sudden bleeding occurs with isolated APTT prolongation, AHA should be suspected, and assays of coagulation factor activity along with inhibitor testing are required.^10)^ Although definitive diagnosis depends on factor activity and inhibitor assays, which typically take 2–5 days, the APTT cross-mixing test can be performed immediately to distinguish factor deficiency from inhibitors such as factor inhibitors or antiphospholipid antibodies, making it a valuable screening tool.^11)^

First-line immunosuppressive therapy for AHA consists of corticosteroids, either alone or in combination with cyclophosphamide. However, rituximab was selected due to hematoma and suspected infection. Rituximab was administered to suppress the factor VIII inhibitor. Corticosteroid therapy was initiated after hematoma removal. NovoSeven was used for bleeding control, followed by emicizumab.

Emicizumab (Hemlibra, ACE910) is a recombinant, humanized bispecific monoclonal antibody that mimics factor VIII activity. Emicizumab has recently attracted considerable attention as a novel therapeutic agent.^12)^ With convenient subcutaneous administration and proven efficacy in the HAVEN 1 study, it is the first prophylactic agent for inhibitor patients.^10,13,14)^ Its pharmacological profile enables prevention of spontaneous bleeding, long dosing intervals, outpatient management, and reduced need for intensive immunosuppression.

This case represents the first report of ICI administration for recurrent malignancy after surgery in a patient with AHA. Although there have been reports of AHA developing after ICI therapy,^15)^ highlighting the need for caution, Mostafa et al.^16)^ previously described a patient with lung adenocarcinoma and AHA who received durvalumab biweekly for 22 weeks, suggesting that ICI use may be feasible with careful monitoring of factor VIII. Similarly, our case indicates that, with referral to hematology and close follow-up, ICIs can be administered during the remission phase of acquired hemophilia, potentially contributing to improved long-term outcomes for patients.

## CONCLUSIONS

AHA is a potentially life-threatening condition that warrants immediate consideration. A thorough diagnostic evaluation, including the APTT cross-mixing test, should be promptly undertaken. While ICIs represent powerful therapeutic agents in oncology, they have been implicated in the development of AHA. This case, however, indicates that in the setting of cancer recurrence, careful surveillance of inhibitor levels during ICI therapy may contribute to achieving favorable long-term outcomes.

## DECLARATIONS

### Availability of data and materials

The datasets generated and/or analyzed during the current study are available from the corresponding author upon reasonable request.
